# Heat Transfer in Cassava Starch Biopolymers: Effect of the Addition of Borax

**DOI:** 10.3390/polym13234106

**Published:** 2021-11-25

**Authors:** Adriana Paola Franco-Bacca, Fernando Cervantes-Alvarez, Juan Daniel Macías, Joan Alexis Castro-Betancur, Reynell Junior Pérez-Blanco, Oscar Hernán Giraldo Osorio, Nayda Patricia Arias Duque, Geonel Rodríguez-Gattorno, Juan José Alvarado-Gil

**Affiliations:** 1 Departamento de Física Aplicada, Centro de Investigación y de Estudios Avanzados del IPN, CINVESTAV-Unidad Mérida, Carretera Antigua a Progreso Km. 6, Merida 97310, YU, Mexico; adriana.franco@cinvestav.mx (A.P.F.-B.); jdanielmacias@yahoo.es (J.D.M.); 2 Departamento de Ingeniería Química, Facultad de Ingeniería y Arquitectura, Universidad Nacional de Colombia-Sede Manizales, Kilómetro 9 vía al aeropuerto, La Nubia, Manizales 170003, Colombia; joaacastrobet@unal.edu.co (J.A.C.-B.); rjperezb@unal.edu.co (R.J.P.-B.); 3 Departamento de Física y Química, Facultad de Ciencias Exactas y Naturales, Universidad Nacional de Colombia-Sede Manizales, Kilómetro 9 vía al aeropuerto, La Nubia, Manizales 170003, Colombia; ohgiraldoo@unal.edu.co; 4 Facultad de Ciencias e Ingeniería, Universidad de Boyacá, Carrera 2ª Este No. 64–169, Tunja 15001, Boyacá, Colombia; npariasd@gmail.com

**Keywords:** cassava starch biopolymer, thermal conductivity, cross-linking, borax

## Abstract

In recent years, polymer engineering, at the molecular level, has proven to be an effective strategy to modulate thermal conductivity. Polymers have great applicability in the food packaging industry, in which transparency, lightness, flexibility, and biodegradability are highly desirable characteristics. In this work, a possible manner to adjust the thermal conductivity in cassava starch biopolymer films is presented. Our approach is based on modifying the starch molecular structure through the addition of borax, which has been previously used as an intermolecular bond reinforcer. We found that the thermal conductivity increases linearly with borax content. This effect is related to the crosslinking effect that allows the principal biopolymer chains to be brought closer together, generating an improved interconnected network favoring heat transfer. The highest value of the thermal conductivity is reached at a volume fraction of 1.40% of borax added. Our analyses indicate that the heat transport improves as borax concentration increases, while for borax volume fractions above 1.40%, heat carriers scattering phenomena induce a decrement in thermal conductivity. Additionally, to obtain a deeper understanding of our results, structural, optical, and mechanical characterizations were also performed.

## 1. Introduction

Since the last century, polymers synthesized from inorganic and organic raw materials like oil, coal, and fossil gas, have encountered several applications in different industries such as agriculture, packaging, furniture, food, electronics, and construction, among many others [1]. These materials represent a huge environmental problem due to the long time required for degradation and its influence on the increasing global warming problem. The availability of polymers which are ecologically friendly and can degrade quickly is necessary and fundamental to reducing plastic waste and improving environmental conditions [2,3]. Nowadays, the synthesis of polymers that suffer significant structural modifications, mainly molecular weight reduction, when placed in interaction with the environment through photo or biodegradation, is currently under study [4,5,6]. On the other hand, biodegradable polymers (biopolymers) have shown the ability to decompose through natural and biological processes in non-toxic carbonaceous soils in an average period of 5 years [7].

In the field of biopolymers, starches account for much of the current research because they come from low-cost, renewable, biodegradable, and recyclable sources. Moreover, they are suitable for different thermal plasticization processes [8,9,10]. However, their commercial application is highly limited due to their low glass transition temperature (*T_g_*) and the lack of relaxation, resulting from the starch molecular chaining and the migration of plasticizers to the environment [11,12]. Authors such as Thitipraphunkul et al. [13], Parra et al. [14], Petersson and Standing [15], Nashed et al. [16], Fama et al. [17], among others, have carried out studies using a wide variety of plasticizers such as sugar, polyalcohols, amino acids, lipids, sorbates, and phosphates with evident structural changes. All of them show that depending on the plasticizer used, starch biopolymers present diverse changes in their mechanical properties.

Structural modifications caused by the addition of plasticizers on starch biopolymers also affect heat transport. In general, polymers have a very low thermal conductivity (0.1–1 W/m·K) [18], due to their intrinsic atomic structural disorder and weak intermolecular interactions [19]. In overcoming this characteristic, starch-based biopolymers hold good prospects because they have donor and acceptor groups of hydrogen bonds [20,21,22], particularly those films and plasticizers with many hydroxyl groups. Hydrogen bonds limit the bond rotation of the polymeric chains and aid the coupling between them, acting as “thermal bridges”, resulting in a continuous thermal conduction network [23,24], which can improve heat transfer and allow the tuning of thermal properties.

Borax is commonly used as a crosslinking agent between the principal polymeric chains in several polymers. Particularly in starch biopolymers, borax acts as an intermolecular bond reinforcer, introducing covalent bonds that complement the natural hydrogen bonding [25]. This characteristic helps to improve the mechanical and solubility resistance, thermal stability, and enhance the uniformity of the molecular structure in the films [26,27]. Polymeric films with borax added show great applicability in the food and drug packaging industry, as they have antimicrobial and texturizing properties [28]. Boron, as a food additive in these kinds of films, does not reach the allowed daily intake, so it cannot be considered harmful [29]. Improving the control on heat transfer is crucial in the food packaging industry, where temperature must be kept stable. A proper choice of the thermal properties is helpful in food preservation, especially in case of perishables such as fruits and vegetables. Starch biopolymer films with borax also present the advantage of acting as protective barriers against bacteria and fungi due to their antimicrobial and fungicidal properties [30].

In this work, the influence of borax on the thermal conductivity of cassava starch biopolymer is studied. We found that the thermal conductivity grows substantially with the increase in borax content, reaching a saturation value at a volume fraction of 1.40% and decreasing for higher borax concentrations. These changes are due to the modifications in the polymeric chain structure, induced by the borax acting as a bonding reinforcer. Our analysis suggests that heat transport is more efficient as the volume fraction of borax increases, because the enhanced crosslinking also induces that the polymeric chains can come closer, generating a better stacked network for heat conduction. For borax concentrations above this saturation limit, heat carrier scattering phenomena appear, producing a decrement in thermal conductivity. To get a deeper understanding of the borax role on the cassava starch polymeric matrix, the characterization of the structural, optical, and mechanical properties was also performed.

## 2. Materials and Methods

Cassava starch biopolymer films were synthesized through the casting method. A homogeneous solution of 5.00 g of sorbitol (98% Merck, Darmstadt, Germany) diluted in 60 mL of distilled and deionized water (DDW) was mixed with a 4.5 g blend of refined food grade cassava starch and sodium tetraborate (borax—99.5% Merck). The cassava starch was purified following the precipitation method proposed by Chiou et al. [31]. Sorbitol was used as a plasticizer because it improves the mechanical properties and facilitates the crosslinking between the principal starch polymer chains [32,33,34,35]. Nevertheless, the amount of sorbitol in all our samples was kept constant to focus our analyses only on the changes produced by the addition of borax [36].

This mixture was heated and stirred at 40 °C and 400 rpm for 10 min to homogenize it. Then, the temperature was increased at increments of 5 °C/min until reaching the cassava [36] starch gelation point at 80 °C. Finally, the resulting solution was left to cool down to room temperature, and 25 mL were poured out into a nonstick plastic container and left to dry at 18 °C and 50% of relative humidity for three days. Seven different borax-to-cassava starch ratios were tested, ranging from 0 to 2.00% volume fraction (% vf), following the same procedure. Samples in the form of membranes with an average thickness of 150 ± 10 microns were obtained. The films were not subjected to any additional treatment previous to their characterization. However, they were stored in sealed containers and maintained at constant humidity and temperature.

The thermal conductivity was determined through the thermal diffusivity. Both properties are related according to *k = αρC_p_*, where *k* is the thermal conductivity, *α* is the thermal diffusivity, *ρ* is the density, and *C_p_* is the heat capacity, all of them at room temperature. To obtain *α*, photothermal radiometry in the transmission configuration was used [37]. The sample was illuminated with a 500 mW laser beam at 635 nm, varying the modulation frequency from 10 to 50 Hz. Each sample was measured five times to obtain a proper thermal diffusivity average value. The films densities (*ρ*) were estimated using the classical method of Archimedes, and the obtained average value was 1100 Kg/m^3^. The *C_p_* and the glass transition temperature were characterized with differential scanning calorimetry (M-DSC Discovery, TA Instruments, New Castle, DE, USA). X-ray diffraction analysis (Bruker D-8 Advance) in Bragg–Brentano configuration with CuK_α_ radiation (*λ* = 1.5418 Å) at 40 kV and 15 mA, was performed to analyze the crystallinity changes resulting from the borax addition. The relative crystallinity percentage was estimated using the method proposed by Lopez-Rubio [38]. UV-Vis characterization was performed in the range from 300 to 700 nm (AVANTES model AVA-Spec 2048, Artisan Technology Group^®^, Champaign, IL, USA). Raman (WITec Alpha-300R, WITec Wissenschaftliche Instrumente und Technologie GmbH, Ulm, Germany) and Infrared (FT-Agilent Cary 630, Santa Clara, CA, USA) spectroscopies were used to analyze molecular interactions and elucidate the changes over the molecular structure related to the borax addition and their relationship with the thermal conductivity. Finally, mechanical testing (Shimadzu EZ-L, 1 kN, Tokyo, Japan) was performed following the ASTM D882-02 (*ASTM D 6400-99 (1976) Standard Specification for Compostable Plastics, Annual Book of Standards. ASTM, Philadelphia*, n.d.) procedure. Measurements of the maximum stress and strain at constant velocity (2 mm/min) were performed, which allowed to observe, at a macroscopic level, the borax effect over the biopolymer structure, to have a deeper understanding of the influence of this cross-linking agent.

## 3. Results and Discussion

**Figure 1:**
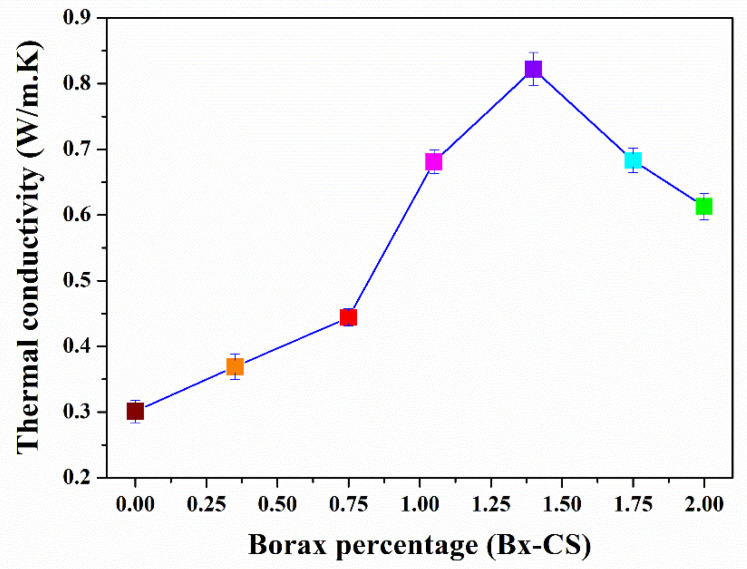
Thermal conductivity as a function of the borax added in cassava starch biopolymer films.

The thermal diffusivity (α), heat capacity (*C_p_*), thermal conductivity (*k*), and glass transition temperature (*T_g_*) of our samples are reported in Table 1. Hereafter, the samples are marked as #Bx-CS, with #Bx for the percentage of added borax and CS for Cassava Starch. The sample without any borax added is referred to as CS. The thermal properties k, α, and *C_p_* were characterized at 28 °C. Figure 1 shows the thermal conductivity of cassava starch films as a function of the borax added. The thermal conductivity of CS is 0.31 W/m·K, a value very similar to those reported for water-soluble polymers with a large number of hydroxyl groups as PVA, PAP, and PAA [31,32]. We found a maximum increment in thermal conductivity of 2.7 times in sample 1.40Bx-CS compared to the CS sample. Above this concentration, the thermal conductivity gradually decreases as the amount of added borax increases.

**Figure 2:**
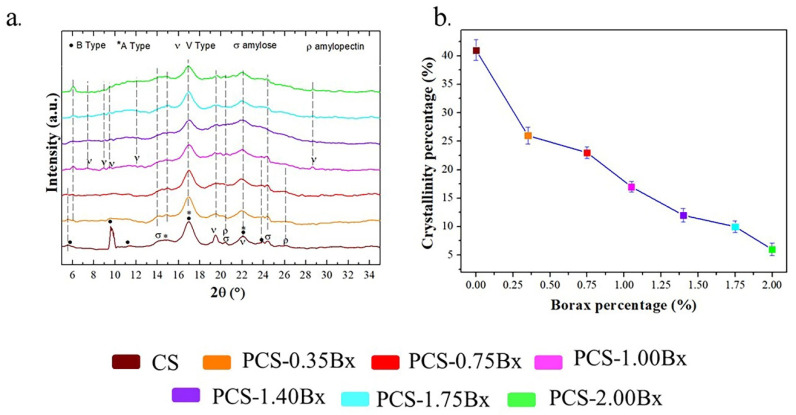
(**a**). X-ray samples’ diffractograms used to calculate the crystallinity percentage showed in (**b**).

Figure 2a shows the X-ray diffractograms for the investigated films. There are diffraction peaks located at 5.62°, 9.67°, 11.46°, 14.27°, 16.92°, 19.53°, 20.45°, 22.12°, 23.98°, 24.45°, and 26.21° for CS film. Type B Starch was identified by the presence of diffraction peaks located at 5.62°, 9.67°, 11.46°, 16.92°, 22.12°, 23.98°. Diffraction peaks located at 14.27°, 16.92°, 23.98° are characteristic of Type A Starch [39]. Diffraction peaks at 7.37°, 8.93°, 9.54°, 12.00°, 19.53°, 22.12°, and 28.64° can be also assigned to V starch polymorph [38]. Diffraction peaks for isolated chains of amylose and amylopectin were detected at 14.27°, 20.45°, 24.25°, and 26.21° [40]. The diffraction pattern for borax–starch systems shows changes in the starch polymorph composition as borax is added. The diffraction pattern for 0.35Bx-CS shows an additional diffraction peak located at 5.99°, its intensity increases with the borax content, this peak can be associated with a less hydrated structure due to borax addition.

Figure 2b shows the crystallinity percentage of our samples calculated from the diffractograms in Figure 2a. Interestingly, but in contrast to what is observed in thermal conductivity, up to 1.40Bx-CS sample, the crystallinity percentage decreases as the amount of borax added increases. Typically, better thermal conductivity could be associated with higher crystallinity. The degree of crystallinity has a significant influence on hardness, density, transparency, and diffusion. However, the properties are not only determined by the degree of crystallinity, but also by the size of the structural units or the molecular orientation. In our samples, particularly in CS, which is the one with the highest crystallinity percentage, the crystalline regions are randomly distributed and isolated from each other, therefore contributing in a limited way to the enhancement of the thermal conductivity [41]. As the amylopectin polysaccharide is the main one responsible for crystallinity in cassava starch [17,42], we envisage that the borax addition induces a decrease in the crystallinity percentage but also allows the formation of bindings between the principal starch polymer chains, causing an improvement in the heat flow. Additionally, the cross-linking reduces the molecular mobility [33], which is evidenced by the increment in the glass transition temperature presented in Table 1.

Our results (Figure 1) show a linear increment of the thermal conductivity related to the increment in cross-linking, similar to the molecular behavior occurring between two polymeric chains with abundant O–H groups [43]. Particularly, Rashidi et al. [44] reported a molecular dynamic analysis of thermal conductivity between a couple of PPA chains, varying the cross-linking percentage through CH_2_ groups. Their simulations show a linear increment in thermal conductivity up to a cross-linking of 60%. After this percentage, a steepening in the slope is present. Our results behave as predicted by Rashidi et al. simulations. We observe a linear increment in thermal conductivity until the sample 0.75Bx-CS, with a slope steepening from here up to 1.40Bx-CS (for 0.75 to 1.40 borax percentage). This behavior is related to the successful bonding between the polymer film structure and the borate ion, resulting in an enhancement in the heat transport through a larger cross-linking [45]. The subsequent decrease in thermal conductivity at higher borax concentrations and an interpretation of our results are presented below in terms of molecular vibrations of the film structure.

**Figure 3:**
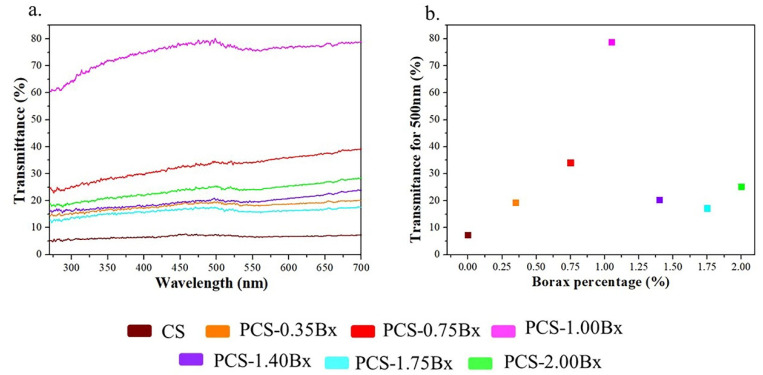
(**a**) Evolution of the UV-Vis transmittance spectra for samples with different borax contents and (**b**) transmittance at 500 nm.

Figure 3a presents the UV-Vis transmittance spectra of the colorless biopolymer films, and Figure 3b shows the transmittance at 500 nm to highlight the changes in the transmittance percentage. Note that no electronic absorption is expected in starch; therefore, the transmittance spectra are essentially dominated by light scattering from macromolecules. The structural reorganization promoted by the borax-assisted cross-linking between polymer chains in the films appears to reduce the size of the macromolecules, reducing the light scattering component. For 1.4% and higher percentages of borax, the transmittance decrement is probably due to agglomeration phenomena which increases the light scattering (see Figure 3b). The slope in the transmittance starting at 300 nm for all samples with borax added (see Figure 3a), results from the formation of polyborates [46].

**Figure 4:**
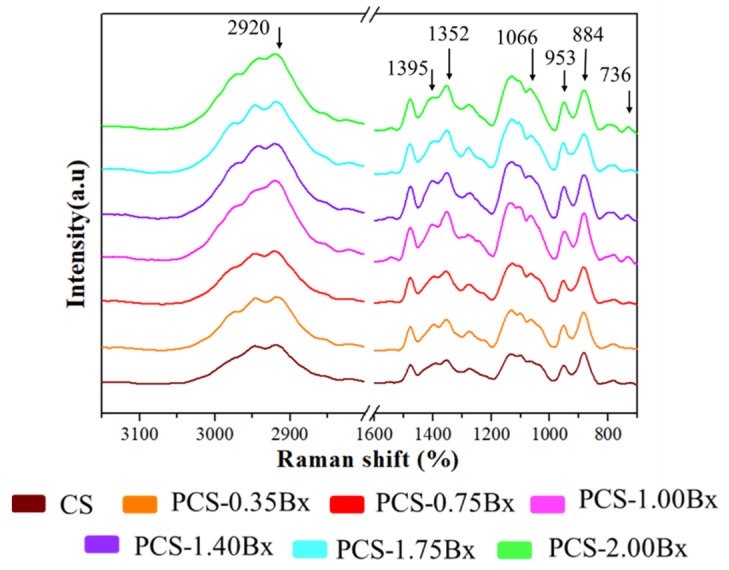
Evolution of the Raman spectra for cassava starch biopolymer films with different borax content.

Molecular vibrational analysis through Raman spectroscopy helps us to interpret, in a qualitative way, the relation between the molecular structure of the biopolymer films and the observed changes in heat transport. Figure 4 shows the Raman spectra from biopolymer films with different volume fractions of borax added. The peaks at 1395 and 1352 cm^−1^ correspond to the B–O–C asymmetric stretching vibration, showing the presence of two complexes formed between the starch biopolymer chains and the hydroxyl-borax group dissociations. Monoborate ions are responsible for the increase in cross-linking through the formation of di-diol tetrahedral complexes with the diol units of the polymeric chains of starch. On the other hand, the boric acid derived from the hydrolysis of borax forms complexes with diol groups of only one polymer chain. Due to lack of space, these latter complexes cannot cross-link polymer chains [47]. The increasing intensity of peak at 1352 cm^−1^, as the amount of borax added increases too, is a consequence of a greater bond density between boric acid and a principal starch polymer chain. The same behavior is observed in the peak at 1395 cm^−1^, which is related to the cross-linking between the principal starch polymer chains, due to the di-diol tetrahedral complexes. This increasing trend stops in the sample 1.40Bx-CS, in the same way as the thermal conductivity does. The observed increase in the peak at 736 cm^−1^ on the sample 1.00Bx-CS and the others with a higher amount of borax correspond to bending vibrations from B–O–H groups. The same behavior is observed in the peaks at 953 cm^−1^ and 884 cm^−1^ due to the presence of unreacted borax and to the borate ion ([B(OH)_4_]^1−^) in the film [48,49]. The bands at the lower wavenumbers correspond to starch glycosidic bonds [50] and vibrations from the glucose–pyranose ring. Finally, the band at 2920 cm^−1^ is produced by hydrogen bonds redistribution in polycomplexes interacting with the ion [B(OH)_4_]^1−^. This increment is remarkable in sample 1.00Bx-CS and the others with higher borax volume fractions. From the behavior of the last band, it is possible to infer that the cross-linking of the principal polymer chains is almost at its maximum level, which agrees with the thermal conductivity characterization.

**Figure 5:**
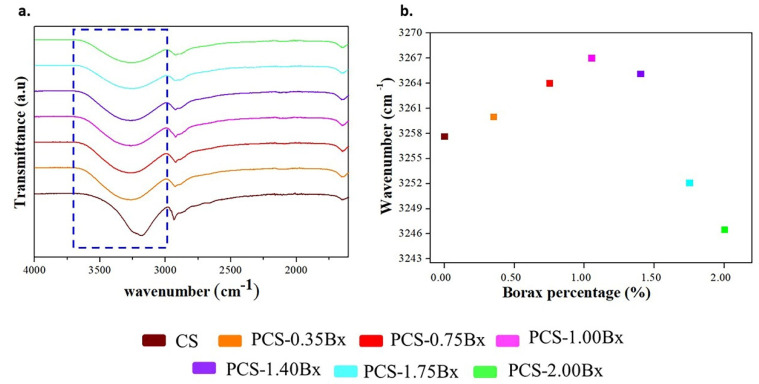
(**a**) FTIR spectrums of cassava starch biopolymer films with borax added. (**b**) Shifting of the band corresponding to the OH bonds shown in (**a**).

Figure 5a shows the FTIR spectrums from cassava starch biopolymer films. This characterization technique allowed us to perform a qualitative analysis of the change in molecular interactions through the band located at 3200 cm^−1^, which corresponds to the stretching of the O–H bonds [51]. The density of hydroxyl groups grows as the added borax does too. The broadening of the peak on the samples with borax added is related to the formation of hydrogen bonding between the principal polymer chains through the ion [B(OH)_4_]^1−^ and the decrease of the initial covalent character.

Figure 5b shows the shifting at higher wavenumbers of the O-H band as the volume fraction of borax added increases up to 1.00%, which is related to the new molecular interactions affecting the inter/intra hydrogen bonds in the starch–sorbitol mixture. The broadening of the peak results from the dispersion in the vibration frequencies allowed in the biopolymer structure. The hydrogen bonds also promote the confinement of the polymer chains, avoiding their rotation and improving the formation of thermal bridges that enhance heat transport [22,50]. In the sample with a 1.40% volume fraction of borax and the others with higher content, the O-H band shifts to lower wavenumbers, due to the formation of borate ion complexes and the weakening of the O–H bonds in the biopolymer, or even preventing their formation [45]. This last observation agrees with the increasing intensity of the peak at 1352 cm^−1^ presented in our Raman spectroscopy analysis. From these results, it is possible to infer the importance of hydrogen bonds in the heat transport process. We also found the limit for this improvement, exceeding the amount of added borax is counterproductive, as can be observed by the decrease in thermal conductivity shown in Figure 1.

**Figure 6:**
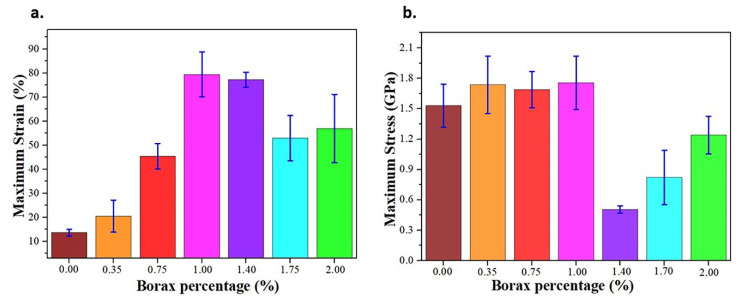
Sample’s maximum strain (**a**) and maximum stress (**b**) measured at constant velocity configuration (2 mm/min).

Figure 6 shows the results of the mechanical tests. The maximum strain and stress are taken at the point where the samples start to break or crack at the waist, resulting from the tensile test. The maximum strain value (Figure 6a) grows as the quantity of borax added increases up to 1.00%. This result is a consequence of the plasticizing effect of boric acid, allowing an increase in the maximum strain [52]. Raman spectroscopy results showed an increase of the peak at 1395 cm^−1^ in the samples up to the 1.00 Bx-CS, proving that the O–H interactions are predominantly electrostatic, enabling the sliding of the principal polymer chains, which in turn gives a large elasticity to the biopolymer films [33,34]. In the samples with higher borax concentrations, the presence of the tetra-coordinated borate ion, acting as a crosslinker and having a stronger bond, generates a decay in the maximum strain. The maximum stress (Figure 6b) is similar in all the samples up to the one with 1.00% of borax added. In these samples, the hydrogen bond interactions, with a great variety of orientations and produced by the boric acid, predominate. Its electrostatic characteristic might favor the heat flow but not necessarily enhance the strength of the biopolymer film. For the concentration of 1.40%, the coexistence of hydrogen bonds from boric acid and the crosslinking from the tetra-coordinated borate ion, which brings closer together the polymeric structure, allows the heat flow to reach its highest efficiency. In samples greater than 1.40%, there is a “competition” between the formation of crosslinking bonds and the plasticizer ones, which is evidenced by the increase in maximum stress, but its value is still smaller than in the sample CS due to the plasticizer effect produced by the borax added [52].

Based on the different characterizations performed to our samples, it is possible to infer that the large quantity of hydroxyl groups that starch has, allows the cross-linking of the principal polymer chains through non-bonding interactions as Van der Waals and by covalent or hydrogen bonds. Together, all of them contribute to the heat flow in biopolymers synthesized from starches [53], acting as “thermal bridges” to form a continuous network for heat conduction and avoiding the scattering of the heat carriers.

The borax addition induces the ordering and approximation among the principal starch polymer chains, which is showed by the UV-Vis transmittance; it is more noticeable in sample 1.00Bx-CS. As shown by Gurau et al. [24], borax also stimulates the formation of hydrogen bonds with an optimal rotation radius that allows the consolidation of a continuous network for heat conduction, taking advantage of the geometrical criterium of linear angles for hydrogen interactions that enhance the heat conduction. In sample 1.40Bx-CS, the space between the principal polymer chains is reduced because of the complete cross-linking of the biopolymer, although for this concentration, the coexistence of the hydrogen and covalent bonds produced by the borax addition takes the thermal conductivity to its highest value. In the samples with greater amounts of borax added, the structuring begins producing clusters of polyborates, which avoids the hydrogen bonding and destroys the continuous heat-conducting network, as is reflected by UV-Vis transmittance, Raman and FTIR spectroscopies. As a result, the thermal conductivity decreases by the generation of scattering centers for the heat carriers, hindering the heat flow, as is shown in Figure 1.

## 4. Conclusions

In this work, the possibility of tuning the thermal conductivity of biopolymer films synthesized from cassava starch is reported. This occurs due to the addition of borax, acting as a cross-linking agent, and generating a continuous network for the heat carriers by limiting the mobility of the free polymer chains. This effect was verified by the increment of the glass transition temperature as the amount of borax added is also increased. It is shown that for samples with 1.40% in volume fraction of borax added, the thermal conductivity value increases 2.7 times compared to the biopolymer film without borax. It is also shown that further increase in borax content induces a detriment in thermal and mechanical properties.

Our results are consistent with the formation of a continuous heat-conducting framework for the heat carriers, emphasizing the importance that hydrogen bonds have over the structuring of the heat-conducting network. Their coexistence and balance along with covalent bonds take the thermal conductivity to its highest value in the system.

We also found that despite the loss of crystallinity, the thermal conductivity was improved, which opens the possibility to design and study materials where, even though the initial crystallinity is not preserved, heat transfer can be improved. Our results establish an upper limit or saturation limit to the borax addition, where the biopolymer structure reaches the maximum benefits from cross-linking. Adding more borax is counterproductive since some of the thermal bridges get interrupted, and the oversaturation of borax generates polyborates, as is observed in Raman spectroscopy. Such oversaturation can partially obstruct the flow of heat carriers by scattering phenomena, inducing a decrease in thermal conductivity.

## Figures and Tables

**Table 1:** Thermal properties of cassava starch films with different percentages of borax added.

Sample	α (×10^−7^ m^2^/s)	*C_p_* (J/g·K)	*k* (W/m·K)	*T_g_* (°C)
CS	1.36	2.22	0.31	33
0.35Bx-CS	1.56	2.36	0.37	38
0.75Bx-CS	2.06	2.15	0.44	55
1.00Bx-CS	2.48	2.74	0.68	83
1.40Bx-CS	3.55	2.31	0.82	88
1.75Bx-CS	3.10	2.20	0.68	139
2.00 Bx-CS	2.90	2.10	0.61	145

## Data Availability

Data is contained within the article.
